# Peritoneal dialysis-associated peritonitis, caused by superior mesenteric artery thrombosis with intestinal necrosis: a case report

**DOI:** 10.1007/s13730-024-00894-y

**Published:** 2024-06-02

**Authors:** Yosuke Osaki, Yujiro Maeoka, Mai Sami, Akira Takahashi, Naoki Ishiuchi, Kensuke Sasaki, Takao Masaki

**Affiliations:** Department of Nephrology, Hiroshima University Hospital, 1-2-3 Kasumi, Minami-Ku, Hiroshima, 734-8551 Japan

**Keywords:** PD-associated peritonitis, Enteric peritonitis, Superior mesothelial artery thrombosis

## Abstract

Peritoneal dialysis (PD)-associated peritonitis is a common complication of PD. Enteric peritonitis is defined as peritonitis arising from an intestinal or intra-abdominal organ source. The delay in the diagnosis or treatment of enteric peritonitis has been reported to increase mortality. Therefore, the early consideration of enteric peritonitis, particularly in cases of culture-negative peritonitis, is imperative. A 67-year-old Japanese man who had been undergoing PD for 3 years, was admitted to our hospital with a diagnosis of PD-associated peritonitis. A month previously, he experienced a bleeding gastric ulcer, which led to severe anemia (hemoglobin 6.3 mg/dL), followed by thrombocytosis. On admission, peritoneal fluid analysis showed a high white blood cell count (WBC: 8,570 /µL), with neutrophils predominating (74.5%). Cultures of both his dialysis effluent and blood were negative. After admission, the WBC count of the dialysis effluent gradually decreased alongside antibiotic therapy, but the patient’s abdominal pain did not improve. After 4 days, enhanced computed tomography showed superior mesenteric artery (SMA) thrombosis and intestinal necrosis. Therefore, emergency intestinal resection and PD catheter removal were performed, and then antithrombosis therapy was initiated. Because the patient’s abdominal pain was improved and platelet count and d-dimer concentration were reduced by these treatments, he was discharged from the hospital after 47 days. Thus, we report a rare case of culture-negative PD-associated peritonitis, which was caused by SMA thrombosis and intestinal necrosis. It is likely that combination of severe calcification of SMA and prolonged thrombocytosis secondary to the severe anemia contributed to the thrombosis.

## Introduction

Peritoneal dialysis (PD)-associated peritonitis is a common complication of PD and is associated with higher healthcare costs and mortality [1–3]. The appropriate treatment for PD-associated peritonitis relies on the identification of the causative organisms in the peritoneal dialysis effluent [4]. Therefore, the International Society for Peritoneal Dialysis (ISPD) guidelines of 2022 recommend reducing the proportion of culture-negative cases to < 15%. However, large variations in the culture-negative proportion (13.4–40%) have been reported [5–7]. Therefore, more careful investigation is required to identify the causes of culture-negative peritonitis to choose the appropriate treatment.

Enteric peritonitis, one of the established categories of PD-associated peritonitis, is defined as peritonitis arising from an intestinal or intra-abdominal organ source, and recognized as one of the conditions that should be considered in the case of culture-negative peritonitis [8]. PD-associated peritonitis owing to some severe diseases, such as pancreatitis, appendicitis, and thrombosis, is included in this category [2, 9–11]. Furthermore, a delay in the diagnosis or treatment of enteric peritonitis has been reported to increase the associated mortality rate by approximately 50% [12, 13]. Thrombosis is one of the key causes of enteric peritonitis, because patients who had decreased kidney function are at a high risk of thromboembolic events [14]. Among the thrombotic diseases, superior mesothelial artery (SMA) thrombosis is difficult to diagnose and represents a serious emergency, with a reported mortality rate ranging from 40 to 80% [15–18].

Thrombocytosis is one of the major causes of the morbidity and mortality associated with thromboembolism [19, 20]. Based on its etiology, thrombocytosis can be divided into two categories: primary thrombocytosis, such as essential thrombocytosis; and secondary thrombocytosis, caused by factors including malignant tumors, inflammation, hemorrhage, and medications [21]. Furthermore, it has been established that primary thrombocytosis increases the risk of thrombosis [20]. However, although secondary thrombocytosis rarely triggers thrombosis, some serious thrombotic diseases, such as abdominal aortic thrombosis and stroke, have been reported to cause secondary thrombocytosis [22–24].

Here, we report a rare case of culture-negative PD-associated peritonitis, which was classified as enteric peritonitis, and caused by SMA thrombosis with intestinal necrosis.

## Case presentation

### Clinical history

A 67-year-old Japanese man who had been undergoing PD for 3 years presented to our hospital with abdominal pain of > 1 week’s duration and a cloudy peritoneal dialysate effluent. He had a history of exit-site and tunnel infection caused by *Streptococcus agalactiae*, and had undergone antibiotic treatment with cefepime and cefazolin and subcutaneous pathway diversion 2 months previously. In addition, he had undergone endoscopic hemostasis and a blood transfusion, owing to severe anemia (hemoglobin concentration 6.2 mg/dL) caused by a bleeding gastric ulcer, 1 month previously. To treat the anemia, ferrous citrate 100 mg/day was administered following the bleeding event, in addition to ferric citrate hydrate 2250 mg/day and darbepoetin 120 µg/month.

### Clinical course

On admission, the patient was 158 cm tall, he weighed 68.5 kg, his body temperature was 36.9 °C, and his blood pressure was 98/62 mmHg. Physical examination revealed abdominal tenderness and swelling, but there were no findings suggestive of the recurrence of the exit site and tunnel infection. Electrocardiography revealed a heart rate of 67/min and sinus rhythm, without any abnormalities. He also had a high white blood cell (WBC) count, a high C-reactive protein (CRP) concentration, mild anemia, and thrombocytosis. Peritoneal fluid analysis showed a high WBC count (8570/µL), with neutrophils predominating (74.5%). Cultures of both the dialysis effluent and blood were negative (Table 1). Plain computed tomography (CT) showed a large amount of intra-abdominal adipose tissue, a typical finding of PD-associated peritonitis, but no findings consistent with a recurrence of the gastric ulcer hemorrhage. On the basis of these findings, we diagnosed PD-associated peritonitis.

**Table 1:** Laboratory results on admission

Parameter	Value	(Normal range)
(Blood)		
Neutrophil	89.5	(38.3–71.1)
Lymphocyte (%)	5.5	(21.3–50.3)
Monocyte (%)	4.8	(2.7–7.6)
Red blood cell (104/μL)	334	(378–499)
Hemoglobin (g/dL)	10	(10.8–14.9)
Hematocrit (%)	30	(35.6–45.4)
Platelet (104/μL)	72	(15.0–36.0)
Aspartate transaminase (U/L)	23	(13–33)
Alanine transaminase (U/L)	30	(8–42)
Lactate dehydrogenase (U/L)	173	(124–222)
Alkaline phosphatase (U/L)	92	(38–113)
γ-Glutamyltransferase (U/L)	8	(13–64)
Total bilirubin (mg/dL)	0.2	(0.4–1.5)
Serum albumin (g/dL)	1.1	(4.0–5.0)
Blood urea nitrogen (mg/dL)	64.6	(8–20)
Creatinine (mg/dL)	9.45	(040–0.70)
Na (mmol/L)	133	(138–146)
K (mmol/L)	3.4	(3.6–4.9)
Cl (mmol/L)	96	(99–109)
Calcium (mg/dL)	9.8	(8.6–10.4)
Phosphate (mg/dL)	4.6	(2.5–4.7)
Uric acid (mg/dL)	3.9	(2.3–7.0)
Fe (µg/dL)	81	(40–188)
UIBC (µg/dL)	13	(112–330)
Ferritin (ng/mL)	676.3	(35.1–353.1)
C-reactive protein (mg/dL)	6.27	(< 0.20)
(Peritoneal dialysis effluent)		
White-cell count (/μL)	8.570	Negative
Neutrophil (%)	74.5	Negative
Eosinophil (%)	0.2	Negative
(Culture)		
Bacterial culture (PD effluent)	Negative	Negative
Mycobacterium culture (PD effluent)	No data	Negative
Blood	Negative	Negative

**Fig. 1:**
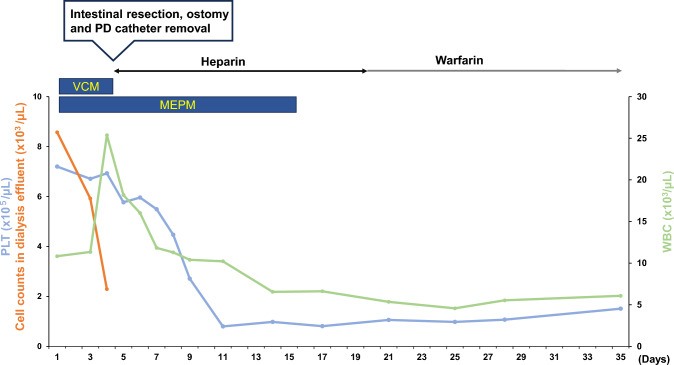
The clinical course of the patient. Changes in the cell counts in the dialysis effluent (orange lines), the white blood cell count (WBC, yellow lines), and the platelet count (PLT, blue lines). MEPM: meropenem, VCM: vancomycin

**Fig. 2:**
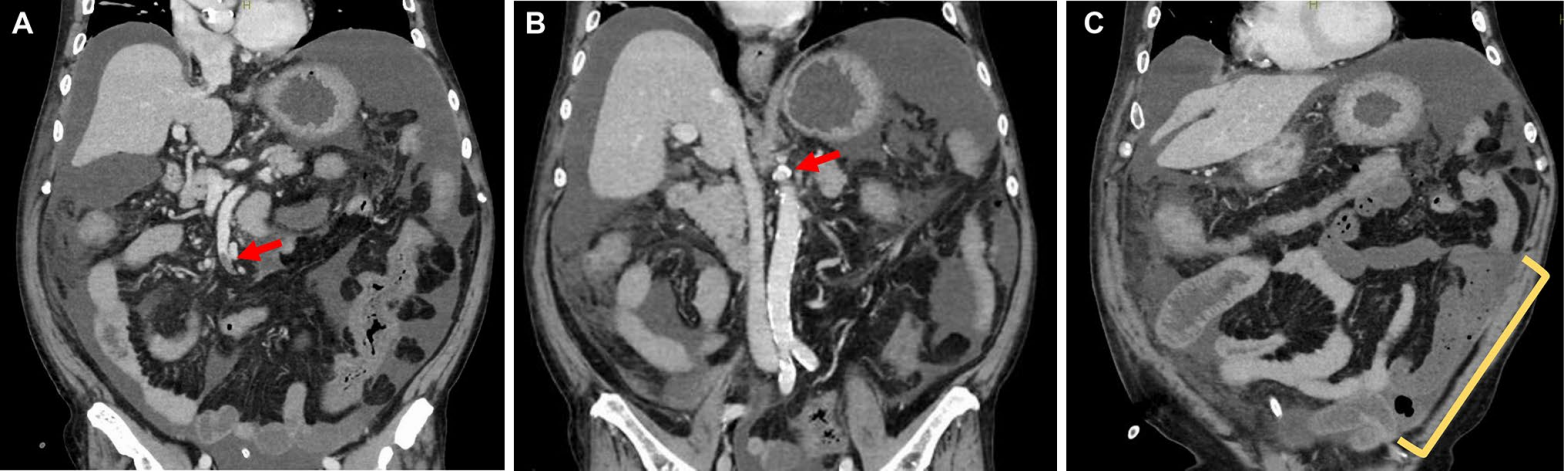
Enhanced computed tomography (CT) findings. Enhanced CT revealed thrombosis of the superior mesenteric artery (SMA) (**A**), calcification of origin of SMA (**B**) and intestinal necrosis (**C**)

We administered 0.5 mg/day of meropenem (MPEM) and 1.0 mg of vancomycin (VCM) intravenously to treat the PD-associated peritonitis (Fig. 1), and the cell counts in the dialysis effluent gradually decreased alongside the antibiotic treatment but there was no amelioration of the patient’s abdominal pain. After 4 days of administration, laboratory testing revealed a high WBC count (25,370/µL) and a high D-dimer concentration (7.5 µg/dL). To differentiate PD-associated peritonitis from other diseases and evaluate the effectiveness of treatment, we performed contrast-enhanced CT. Abdominal CT revealed severe calcification of SMA, SMA thrombosis and intestinal necrosis, but no findings indicative of intestinal perforation (Fig. 2). Therefore, intestinal resection, ostomy, and PD catheter removal were performed on the same day. After this surgery, hemodialysis was initiated as a renal replacement therapy, 5000 U/day of heparin was administered as an antithrombotic treatment for 2 weeks, and then this was changed to oral warfarin administration. These treatments ameliorated the patient’s abdominal pain and reduced his platelet count and d-dimer concentration. Because transesophageal echocardiography revealed no obvious cardiac abnormalities, he was discharged from hospital after 47 days.

## Discussion

In this report, we have described a rare case of culture-negative PD-associated peritonitis caused by SMA thrombosis with intestinal necrosis, which was classified as enteric peritonitis. In addition, the combination of severe calcification of SMA and thrombocytosis secondary to the severe anemia triggered by the patient’s bleeding gastric ulcer was considered to be the cause of his thrombosis.

The identification of the causative organism in the dialysis effluent is important for the selection of the most appropriate treatment, and the identification of the underlying primary disease is required in culture-negative cases. In an attempt to identify the causative organism, we repeated the cultures of the patient’s dialysis effluent and blood but we could not detect any bacteria. The most common explanations for culture-negative peritonitis are antibiotic therapy within the preceding 30 days and technical problems during the dialysate culture [6, 7]. The present patient had taken antibiotics to treat his exit-site and tunnel infection, but these had been administered 2 months previously, and, therefore, were not considered to be the cause of the negative culture result. It has been previously reported that culture-negative peritonitis is not associated with higher risks of hospitalization, death, or a change to hemodialysis [25, 26]. However, a delay in the diagnosis or treatment of enteric peritonitis, which is one of the conditions that should be considered in the case of culture-negative peritonitis, has been reported to increase mortality by approximately 50% [2, 12, 13]. Therefore, it is important to evaluate the reasons for culture negativity at an early stage, especially if treatment is ineffective [6, 27].

For the evaluation of the effectiveness of treatment, the WBC count of the dialysis effluent and the clinical symptoms should be evaluated longitudinally. Appropriate antibiotic treatment generally ameliorates the symptoms of patients within 72 h, but a WBC count in the dialysis effluent of > 1090/µL 2 days following the initiation of treatment is a prognostic marker of treatment failure [2, 28, 29]. The present patient was initially treated empirically with antibiotics, and his dialysis effluent WBC count slowly decreased with antimicrobial therapy and peritoneal lavage, but remained > 2000/µL 4 days after the initiation of treatment. Moreover, his abdominal pain had not been ameliorated. In cases of inadequate response to antimicrobial treatment, eosinophilic peritonitis should be considered [30]. In this case, we ruled out the eosinophilic peritonitis because there was no increase of eosinophils in PD effluent throughout the clinical course. This clinical course and the findings of enhanced CT confirmed that the antibiotic treatment had not been effective and that the PD-associated peritonitis was non-infectious and associated with SMA thrombosis and ischemic enteritis. In addition to CT imaging, the findings of PD effluent may be helpful in the early diagnosis of enteric peritonitis. It is reported that amylase concentrations is increased in PD effluent from enteric peritonitis including ischemic enteritis [31]. Although we did not measure the amylase concentrations in PD effluent in this case, the measurement should be considered in suspected cases of enteric peritonitis.

We investigated which underlying diseases might have contributed to the SMA thrombosis. Initially, we considered cardiac diseases, including intracardiac thrombi, acute myocardial infarction, and atrial fibrillation, as causes of the thrombosis, because cardiac diseases are reported to be a cause of 70% of cases of SMA thrombosis [18]. However, the present patient did not have arrhythmia or a cardiac disease that could have caused his thrombosis. On the other hand, the CT revealed severe calcification of the SMA, which is reported as a cause of SMA thromboembolism [32]. We considered that the stenosis of SMA contributed to thrombus formation. Additionally, laboratory testing on admission revealed thrombocytosis, which is also known to be associated with thromboembolism [33]. Most cases of thrombosis caused by thrombocytosis are primary thrombosis, although severe thrombosis arising as a result of secondary thrombocytosis has also been documented [20, 22–24, 34]. In the present case, a relationship between the thrombocytosis and hemorrhage was suspected, because the patient’s platelet count increased from 29.5 to 72.0 × 10^4^/µL over the course of the month following the bleeding gastric ulcer. Therefore, we concluded that the combination of severe SMA calcification and secondary thrombocytosis played a role in the development of the SMA thrombosis.

To investigate the cause of secondary thrombocytosis in detail, we assessed the patient’s level of iron deficiency, which is known to be a contributing factor to secondary thrombocytosis [35]. Song et al*.* reported that the risk of thrombosis is twice as high in patients with thrombocytosis and iron deficiency anemia than in those with iron deficiency anemia alone (15.8% *vs*. 7.8%, respectively) [36]. On admission, the present patient showed a high transferrin saturation and a high ferritin concentration, (86% and 676 ng/dL, respectively). Moreover, we could not identify any evidence of iron deficiency nor any indicators of iron-deficiency anemia, such as microcytic anemia, following the bleeding event, despite the presence of severe anemia. One possible reason for this absence of iron deficiency was that appropriate iron supplementation was administered following the bleeding event. Therefore, we concluded that not only the iron-deficiency anemia but also the severe anemia, without iron deficiency, may have contributed to the secondary thrombocytosis.

In conclusion, we have reported a rare case of culture-negative PD-associated peritonitis caused by SMA thrombosis with intestinal necrosis. In cases of culture-negative PD-associated peritonitis, enteric peritonitis caused by arterial thrombosis should be considered at an early stage; and in particular, special attention should be paid to patients who experience bleeding episodes, with or without iron deficiency.
